# Determination of Brain Death/Death by Neurologic Criteria in Adults: A Structured Framework for Medical Educators

**DOI:** 10.1212/NE9.0000000000200316

**Published:** 2026-05-06

**Authors:** Justine Cormier, Sean Marinelli, Catherine S.W. Albin, Nicholas A. Morris, Ariane Lewis, Rachel B. Beekman, David Matthew Greer, Sarah Wahlster, James A. Town

**Affiliations:** 1 Ayer Neuroscience Institute, Hartford Hospital, CT;; 2 Department of Neurology, University of Washington, Seattle;; 3 Department of Neurology, Division of Neurocritical Care, Emory University, Atlanta, GA;; 4 Department of Neurology, Program in Trauma, University of Maryland School of Medicine, Baltimore, MD;; 5 Departments of Neurology and Neurosurgery, NYU Langone Medical Center, NY;; 6 Department of Neurology, Yale University, New Haven, CT;; 7 Department of Neurology, Boston University, MA;; 8 Department of Neurological Surgery, University of Washington, Seattle;; 9 Department of Anesthesiology, University of Washington, Seattle; and; 10 Department of Medicine, Division of Pulmonary, Critical Care and Sleep Medicine, University of Washington, Seattle.

**Figure 1:**
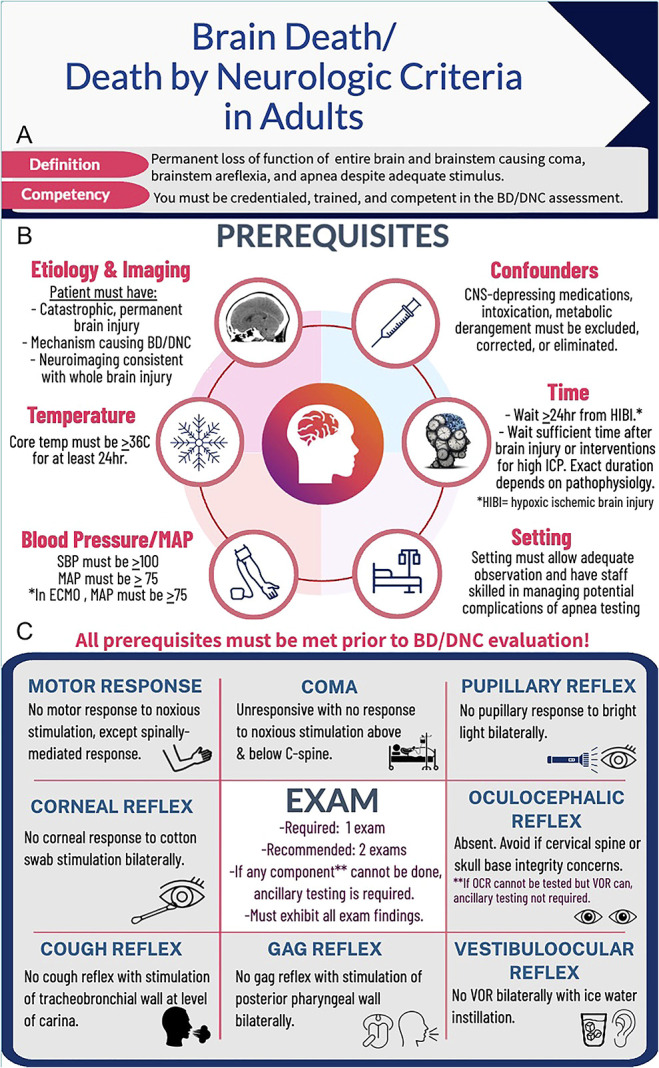
(A) Definition and Competency Requirements; (B) Overview of the Prerequisites for the BD/DNC Evaluation; and (C) Key Components of the Clinical Examination for the BD/DNC Assessment

**Figure 2:**
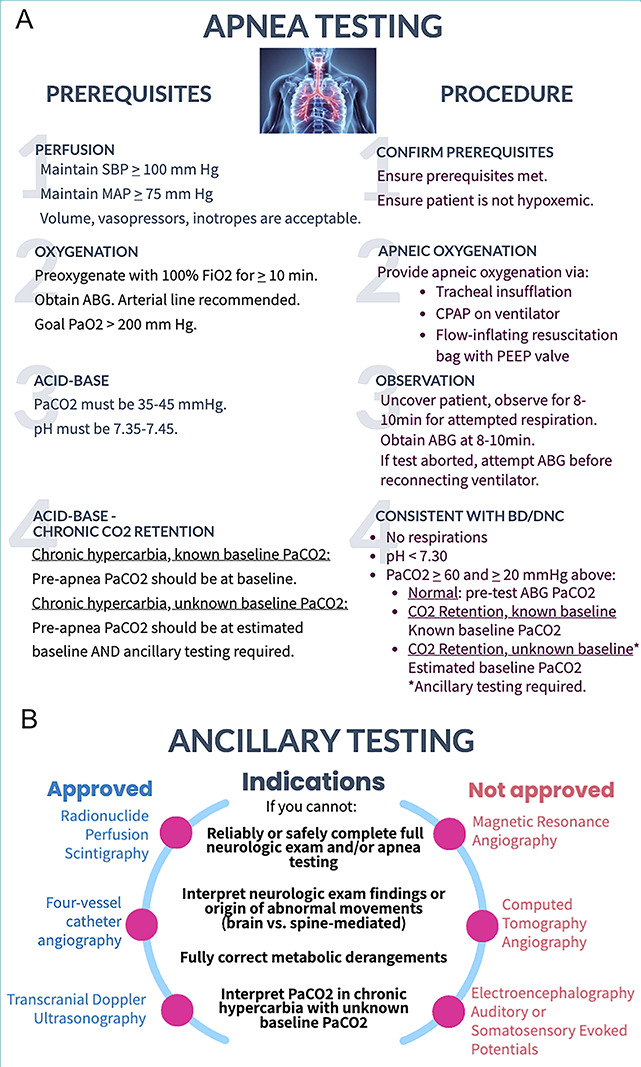
(A) Prerequisites and Procedure for Apnea Testing. (B) Indications for Ancillary Testing and Recommended Ancillary Testing Modalities

Accurate performance of the brain death/death by neurologic criteria (BD/DNC) evaluation is critically important; however, understanding this nuanced process can be challenging. Practice variations have been widely reported.^1^ The 2023 American Academy of Neurology, American Academy of Pediatrics, Child Neurology Society, and Society of Critical Care Medicine guidelines emphasize that clinicians performing BD/DNC evaluations should have specific education and demonstrate competency in performing this assessment to ensure accurate, consistent application of the guidelines. We aimed to create an easily accessible, user-friendly, quick reference guide to complement the guidelines and aid in BD/DNC education.^2^ We present an infographic that provides an overview of the BD/DNC evaluation process, including definitions, prerequisites, clinical examination, apnea testing, and ancillary testing (Figures 1 and 2).
